# Role of Micro RNA-205 in Promoting Visceral Adiposity of NZ10 Mice with Polygenic Susceptibility for Type 2 Diabetes

**DOI:** 10.4172/2155-6156.1000574

**Published:** 2015-06-27

**Authors:** Nikhil Adi, Jennipher Adi, Liliana Cesar, Paul Kurlansky, Arthur Agatston, Keith A Webster

**Affiliations:** 1 Department of Molecular and Cellular Pharmacology, Miller School of Medicine, University of Miami, Miami, FL, USA; 2 Vascular Biology Institute, Miller School of Medicine, University of Miami, Miami, FL, USA; 3 Columbia University, New York, USA; 4 Miami Baptist Hospital, Miami, FL, USA

**Keywords:** Micro-RNA, Visceral adipose, Obesity, 3t3-l1, Adipogenesis

## Abstract

**Scope:**

To characterize diet-dependent miRNA profiles and their targets in the visceral adipose of mice with polygenic susceptibility to type 2 diabetes.

**Methods and results:**

Six-week NONcNZO10/LtJ (NZ10) and control SWR/J mice were subjected to high protein-fish oil or control diets for 19 weeks and micro-RNA microarray analyses were implemented on visceral adipose RNA. We found that 27 miRNAs were significantly induced and 10 significantly repressed in the VA of obese NZ10 mice compared with controls. 12 selected regulated miRNAs were confirmed by RT-PCR based on the microarray data and we demonstrated that the expression of these miRNAs remained unaltered in the VA of control SWR mice. To assess the possible functional roles of miRNAs in adipogenesis, we also analyzed their expression in 3T3-L1 cells during growth and differentiation. This revealed that suppression of miRNA-205 alone correlated selectively with increased cell proliferation and lipid formation of adipocytes.

**Conclusion:**

Diet and genetics control the expression of obesity-regulated miRNAs in the visceral adipose of NZ10 mice.

## Introduction

Micro-RNAs (miRNAs) are short 21–23 nucleotide RNAs that regulate gene expression at the posttranscriptional level [1,2]. Obesity and insulin resistance are key features of type 2 diabetes. In obese individuals, increased adiposity is a consequence of increased number and size of adipocytes. The relative degree of hypertrophy versus hyperplasia influences the level of body fat and the metabolic consequences of obesity [3,4]. The involvement of miRNAs in adipocyte differentiation has been studied using 3T3-L1 cells wherein selective miRNAs have been assigned various roles in pre-adipocyte differentiation [5]. It is now known that several repressor miRNAs implicated in adipogenesis are inversely expressed in the obese adipose tissue [6] and this inverse regulatory pattern has been characterized in mouse models of obesity and in humans [7]. To date microRNA targets have only been identified in monogenic or diet-induced models of obesity and diabetes. These models involve severe obesity and hyperinsulinemia that is usually associated with hyperphagia and extreme leptin levels. There have been no reported studies on the regulation of microRNAs in polygenic models of obesity-induced diabetes.

NONcNZO10/LtJ (NZ10) is a recombinant congenic strain for obesity-induced type 2 diabetes that was developed at The Jackson Laboratory (Bar Harbor, Maine). The polygenic nature with a maturity-onset transition from impaired glucose tolerance to a stable non-fasting hyperglycemia and a relatively mild obesity of the mice closely resembles human T2D. The NZ10 mice differ from the parent NZO/HILt strain in acquiring adiposity primarily in visceral rather than subcutaneous fat depots and this may contribute to the T2D-susceptibility phenotype [8]. Also, NZ10 mice accumulate more visceral fat and intramuscular triglyceride levels relative to wild type C57BI/6J mice subjected to either low or high fat (6 or 11%) diet [9]. We have recently reported that a high protein-fish oil diet prevents the development of obesity and type 2 diabetes in NZ10 mice and also influences visceral adipose remodeling by regulating the expression of matrix metalloproteinases and their inhibitors [10]. Here we report that miRNA-205 is selectively implicated in adipocyte division and lipid accumulation *in vitro* and *in vivo*.

## Methods

### Animals and diets

Four-week old male NZ10 mice and their non-diabetic SWR/J counterparts were purchased from The Jackson Laboratory (Bar Harbor, Maine). After 2 weeks of acclimatization mice were randomized into 2 groups (at least 10 mice per group) to receive control (CD) or High Protein-Fish Oil (HPO) diets for a period of 19 weeks [10]. Blood glucose after 12 hrs of fasting was measured with a glucometer (Freestyle, Abbott Diagnostics). For tissue harvest, mice were fasted for 12-h, sacrificed by cervical dislocation and the visceral adipose depots including epididymal, perirenal, and retroperitoneal rapidly dissected and processed for RNA analysis.

### MicroRNA microarrays

Pooled samples (n=5 per group) were homogenized in Trizol (Invitrogen, Carlsbad, CA) and total RNA was isolated. Quality of the total RNA samples was assessed using UV spectrophotometry and agarose gel electrophoresis. The samples were treated with DNAse and low-molecular weight (LMW) RNA isolated by ultrafiltration through YM-100 columns (Millipore) and subsequent purified using a RNeasy MinElute Clean-Up Kit (Qiagen, Valencia, CA). RNA samples were subjected to custom microarrays containing 374 probes covering mouse mature microRNAs present in Sanger 9.0 mirBASE at Ocean Ridge Biosciences (ORB, Palm Beach Gardens, FL). The species-specific, unmodified oligonucleotide probes are 34–44 bases in length from the Invitrogen Ncode Version 2.0 probe set (Invitrogen, Carlsbad, CA). The microarrays were produced by Microarrays Inc. (Huntsville, Alabama), and consisted of epoxide glass substrates spotted in triplicate with each probe. The LMW RNA samples were 3’-end labeled with Oyster-550 fluorescent dye using the Flash Tag RNA labeling Kit (Genisphere, Hatfield, PA). Labeled LMW RNA samples were hybridized to the MicroRNA microarrays according to conditions recommended in the Flash Tag RNA labeling Kit manual. The microarrays were scanned on an Axon Genepix 4000B scanner, and data was extracted from images using GenePix V4.1 software. The data was preprocessed and normalized as described in Enquobahrie et al. [11].

### Real time PCR

MicroRNA expression levels were assayed by real time PCR using the ABI 7900HT thermal cycler with custom assays (Life Technologies, Grand Island, NY). PCR reactions were conducted at 95°C for 10 min and then followed by 40 cycles (15 s at 95°C, 60 s at 60°C). The following Assay IDs 000587, 000463, 002249, 002299, 000502, 002251, 002300, 000507, 000509, 000546, 001077, 002607, 001670, 001678 and Mm01184322_m1 were used for miRNAs -29c, 141, 143, 191, 200a, 200b, 200c, 203, 205, 335, 429, 665, 685, 691 and PPARγ respectively. Eukaryotic 18S rRNA (Assay ID Hs99999901_s1) for PPARgamma gene expression and snoRNA 202 and 234 (Assay IDs 1232, 1234) for miRNA expression normalization were used.

### Cell culture

Mouse 3T3-L1 preadipocytes were purchased from Zen-Bio (Research Triangle Park, NC) and were cultured in preadipocyte maintenance medium (PM-1-L1, Zen-Bio). For induction of adipogenesis the cultured preadipocytes were grown to 100% confluency and after 2 days the media was replaced with adipogenic differentiation medium (DM-2-L1, Zen-Bio). After 3 days the DM-2-L1 media was replaced by adipocyte maintenance medium (AM-1-L1, Zen-Bio). Adipocytes were fed with AM-1-L1 every 3^rd^ day. Preadipocytes were transfected with pre or anti-miR-205 oligos (Life Technologies, Grand Island, NY) according to manufacturer’s instructions.

### RNA isolation and reverse transcription 3T3-L1 cells

Total RNA was isolated from 3T3-L1 preadipocytes and adipocytes using miRVana kit (Life Technologies, Grand Island, NY). The concentration of RNA was determined by spectrophotometry, using Nanodrop-1000 (Nanodrop Technologies, Wilmington, DE). Reverse transcription for gene expression was performed on 200 ng/μl of RNA with High-Capacity cDNA Reverse Transcription kit using random primers (Life Technologies, Grand Island, NY). Reverse transcription for miRNA expression was performed on 10 ng of RNA with Taqman miRNA reverse transcription kit using custom primers for each miRNA (Life Technologies, Grand Island, NY).

### Cell cycle proliferation

Cell proliferation of pre or anti-miR-205 transfected preadipocytes was measured using cell proliferation ELISA, BrdU colorimetric kit (Roche, Indianapolis, IN) as per manufacturer’s instructions. Briefly, 3T3-L1 preadipocytes were transfected with Pre (10 μM)- or Ant-imiR- 205 (10 μM) along with controls (transfection medium) in a 96 well plate for 48 h followed by incubation with 10uM BrdU for 2.5 h. The cells were then fixed and incubated with Anti-BrdU POD. Incubation with substrate was done for 20 minutes and the reaction was stopped using stop solution. Absorbance was measured using a plate reader at 450 nm.

### Oil red O staining

Preadipocytes were transfected with pre (10 μM) or anti-miR-205 (10 μM) in 6 well plates. Oil Red O staining at Day 3 and Day 7 visualized adipocytes formed. For Oil Red O staining the adipocyte maintenance medium was aspirated and cells were washed 3 times with phosphate buffered saline (PBS) and fixed with 4% paraformaldehyde for 30 minutes. After 30 minutes the paraformaldehyde solution was removed and cells were rinsed 3 times with PBS and twice with water. Oil red O solution was added and the cells were further incubated for 50 minutes. After 50 minutes the stain was aspirated and cells were rinsed 3 times with water. The stained cells were photographed and dye extraction solution was added. The plate was set on an orbital shaker for 20 minutes and the extracted dye was transferred to a 96 well plate and absorbance measured at 520 nm with a plate reader spectrophotometrically. Stain extracted from wells lacking cells represents non-specific binding of the dye to the plate and its value was subtracted from the absorbance of experimental wells.

### Statistical analysis

Data are expressed as mean ± SEM. Differences between two groups were analyzed by Student’s “t” test and Mann-Whitney U test using Graphpad Prism 5.0.

## Results and Discussion

**Figure 1:**
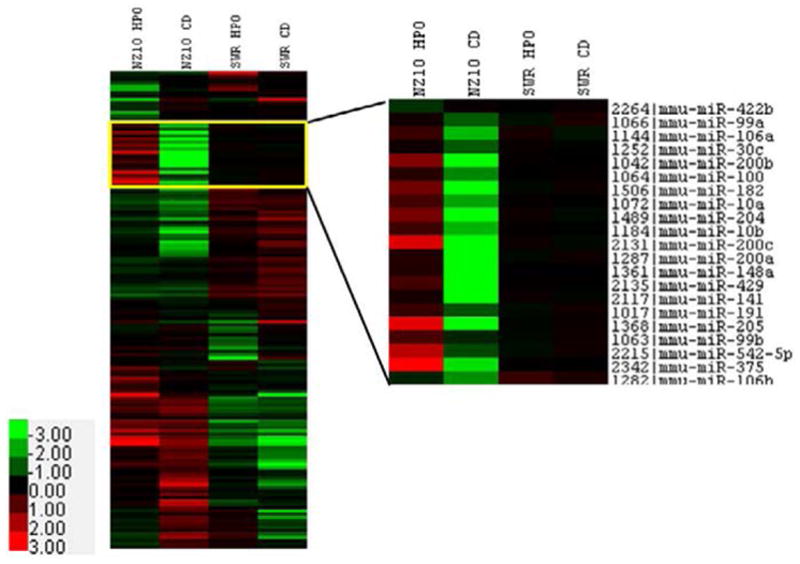
miRNA expression analysis of visceral adipose tissue using microarray analysis. Heat maps show differences in miRNA levels in the VA of NZ10 and SWR mice fed CD and HPO diet for 19 weeks. Inset heat map shows miRNAs significantly up-regulated by HPO diet only in the VA of NZ10 mice and not the SWR mice of same age on either diet.

**Figure 2:**
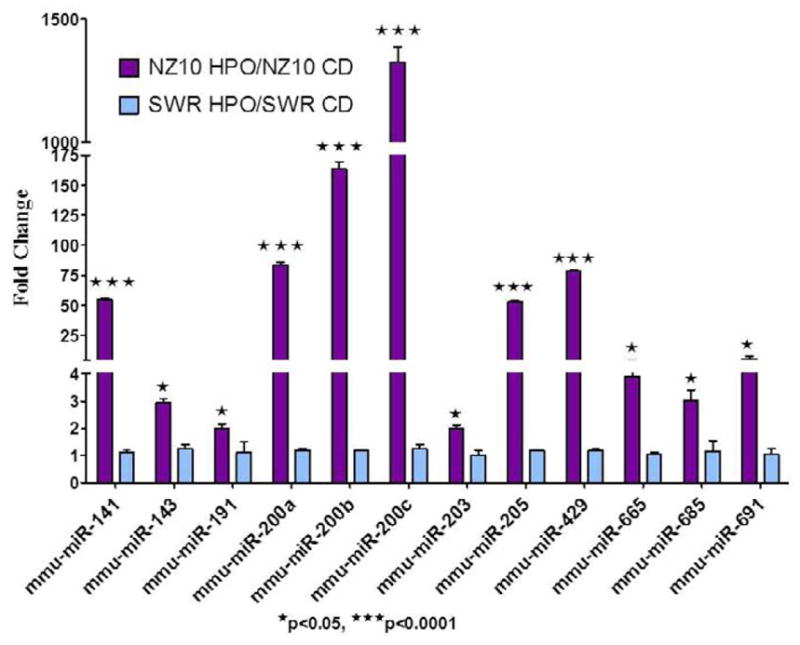
Real time PCR analysis of miRNAs revealed significant up-regulation in the VA of NZ10 mice fed HPO over those fed CD (Purple Bars) and no change in expression of these miRNAs in VA of SWR mice fed any diet (Blue Bars). snoRNA-202 was used as an endogenous control and fold changes were calculated over CD fed mice.

We recently reported that the HPO diet significantly reduced the body weight of NZ10 mice compared with their counterparts on CD or the SWR mice on either diet [10]. NZ10 mice fed CD had increased body fat and displayed hyperinsulinemia and fasting hyperglycemia that was not seen in mice fed HPO or SWR mice fed either diet [10]. These results confirm the development of a diabetogenic phenotype in NZ10 mice fed CD. NZ10 mice may be useful for identifying susceptibility loci and genetic background-diet interactions [12–14]. As a first step towards such analyses we implemented micro-RNA arrays using RNA from the VA of NZ10 and SWR strains fed CD or HPO diets as described [10]. miRNA microarray analysis of the VA revealed 27 miRNAs that were significantly up-regulated and 10 miRNAs that were significantly down-regulated in the NZ10 mice fed HPO diet vs. those fed CD for 19 weeks (Figure 1, Tables 1–2). RT-PCR confirmed increased expression of 12 up-regulated miRNAs in the VA of NZ10 mice fed HPO. Results from RT-PCR analyses of individual RNA samples are in good agreement with the microarrays (Figure 2, Table 1–2). The HPO diet significantly up-regulated the expression of the miR-200 family as well as miRs- 143, 191 and 205 in the VA of NZ10 mice but not in SWR mice suggesting complementary roles for diet and genetics in the regulation of these miRNAs (Figure 2).

Our results on the expression trends of the miR-200 family: miRs- 200a, 200b, 200c, 141 and 429 are consistent with results reported previously using epididymal fat pads of DIO mice fed high fat diet [15]. Whereas, the expression of miR -148b was significantly inhibited in the VA of obese-diabetic NZ10 mice and the levels of miR-379 previously reported to be up-regulated in the adipose of high fat fed DIO mice was also significantly induced in the VA of obese-diabetic NZ10 mice (Table 1). Similarly miRs- 1 and 122a were inhibited in the adipose of DIO mice and showed significant up-regulation in the obese NZ10 mice (Table 2) [15]. Another example of a miRNA showing differential expression patterns in the adipose of obese mice is miR-143. Its expression in the present study was significantly down-regulated in the VA of obese-diabetic NZ10 mice fed CD compared to those fed HPO and unchanged in SWR mice (Figure 2). These results help resolve discrepancies in the literature where miR-143 has been shown to be unchanged [15], decrease [6], or increased [16] in parallel with obesity. The differences could be due to genetic background or different responses of miR-143 in different fat depots.

**Figure 3:**
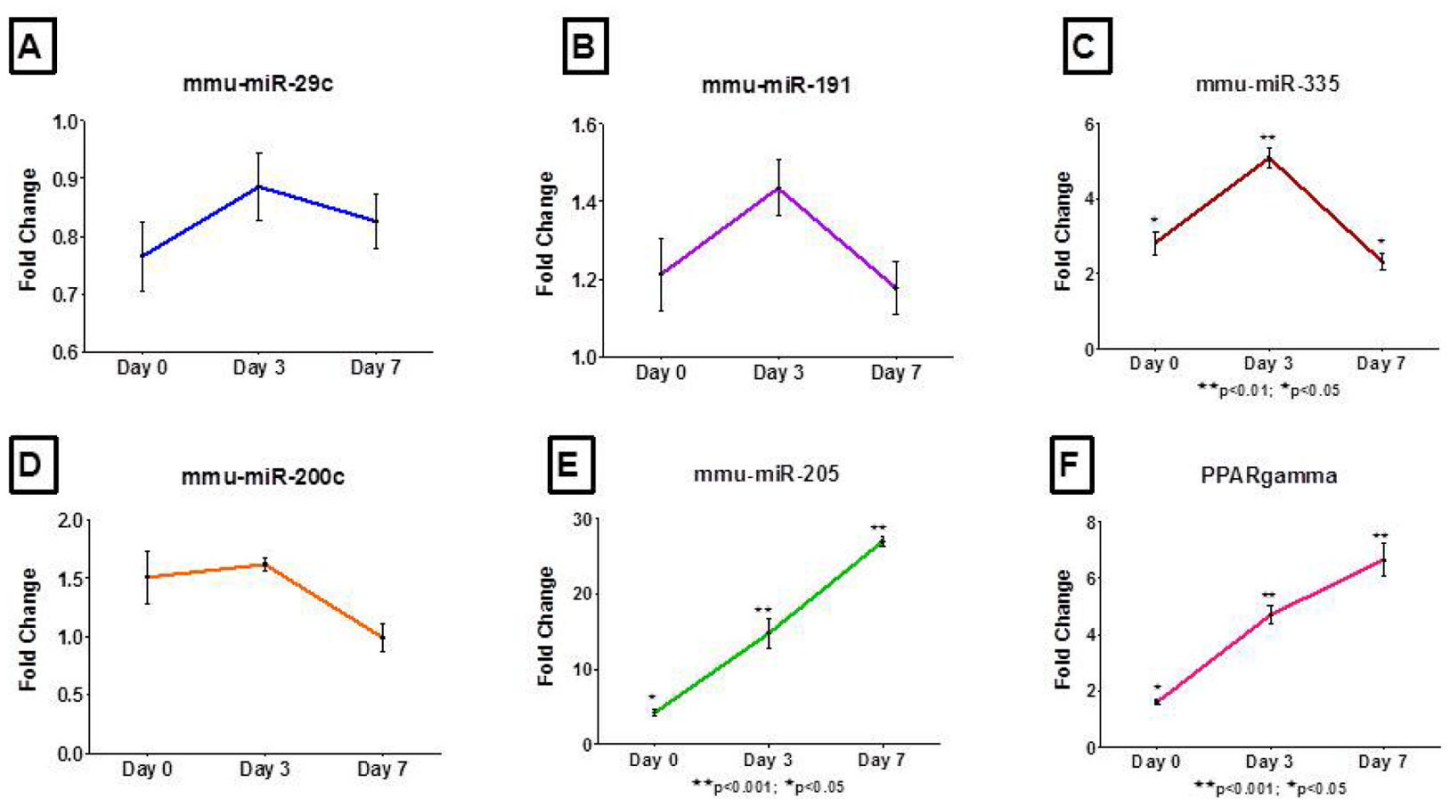
miRNA expression as assessed by real time PCR at Day 0, 3 and 7 of 3T3-L1 preadipocyte differentiation. miRNA expression for Day 0, 3 and 7 are relative to the preadipocyte levels and snoRNA-234 and 18S rRNA were used as endogenous controls for normalization of miRNA and PPARgamma gene expression respectively. A schematic representation describing the 3T3-L1 adipogenesis paradigm for this study is provided in the supplement (Figure 1).

Because members of miR-200 family and miR-205 showed significant inhibition in the VA of obese mice, we chose to evaluate whether any of these miRNAs are implicated in adipogenesis. We quantified the expression of miRs- 29c, 141, 191, 200a, 200b, 200c, 205, 335 and PPARγ in 3T3-L1 cells at different stages of differentiation. MiRs- 200a, 200b and 141 were below levels of RT-PCR detection in 3T3-L1 cells either as preadipocytes or after differentiation into adipocytes. The levels of miRs- 29c, 191 and 200c increased up to Day 3 and then reverted to preadipocyte levels by Day 7 (Figures 3a-3d). The trend was similar for miR-335 expression with a peak induction at Day 3 followed by decline (Figure 3c). MiR-205 was unique insofar as its expression was significantly elevated at all-time points during adipogenesis in parallel with expression of the PPARγ gene (Figures 3e,3d). By integrating separate datasets of genome wide PPARγ binding sites in 3T3-L1 cells John et al. [17] determined that miRs- 103-1, 182/96/183, 205 and 378 are regulated by PPARγ.

**Figure 4:**
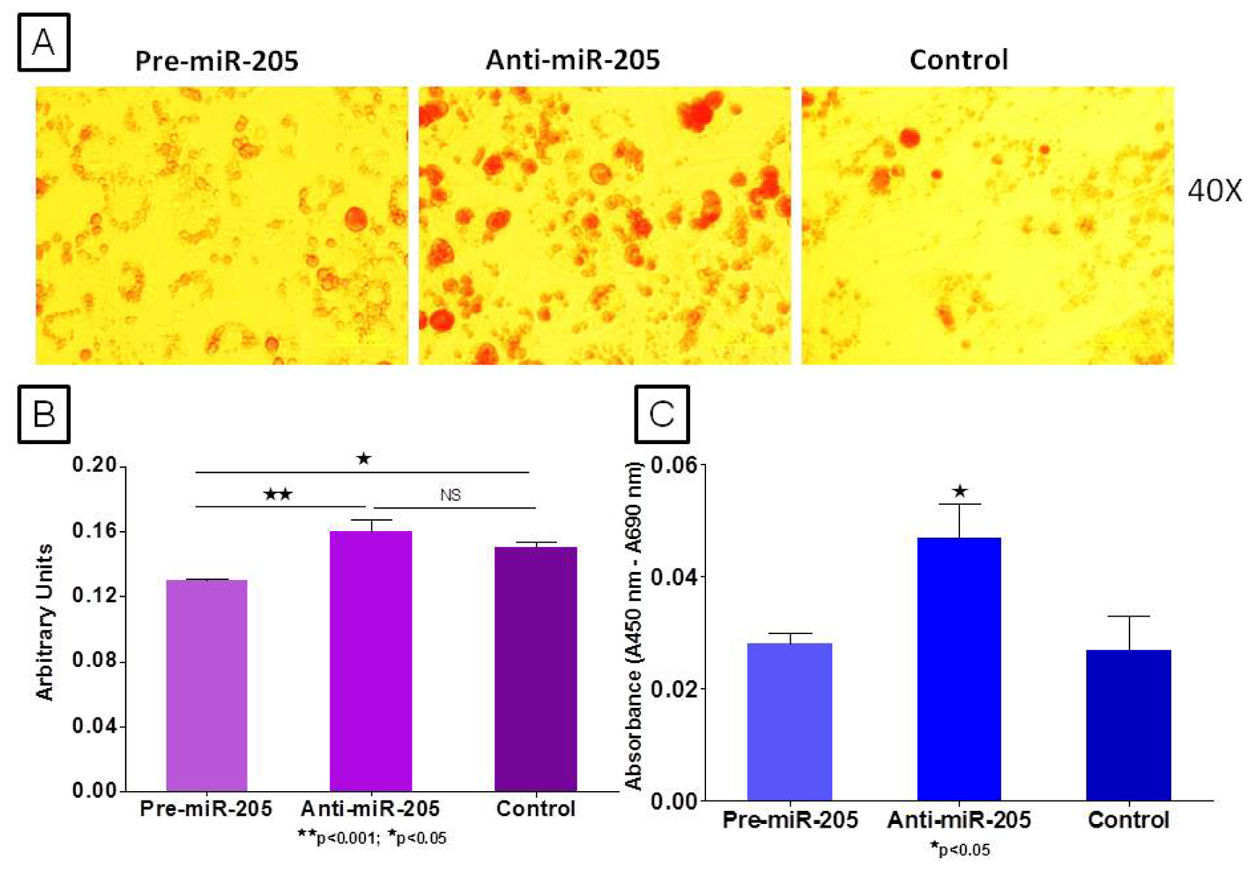
Lipid formation was assessed at Day 3 and Day 7 after miR-205 over-expression and inhibition in 3T3-L1 cells. (A) Oil red O staining at Day 3 revealed significantly increased lipid staining in the miR-205 inhibited cells compared to those over-expressing miR-205. (B) Spectrophotometric quantification of extracted oil red O stain (n=8 each group) at day 3. (C) Cell cycle proliferation was assessed in 3T3-L1 cells 48 hours after transfection with pre and anti-mIR-205 using BrdU elisa (n=8, each group).

Results from our analyses reveal significant inhibition of miRs-182 and 205 in the VA of obese NZ10 mice and increased expression of miR-205 in 3T3-L1 during adipogenesis (Figure 3e) [17]. This is the first study to show inhibition of miRs- 182 and 205 in VA of obese-diabetic mice. The functions of these miRNAs in preadipocytes are still unknown. Because PPARγ levels are not induced in 3T3-1 cells until adipogenesis is induced [18] we chose to assess the role of miR- 205 independently of PPARγ in the preadipocyte stage. To this end we modified miR-205 expression in 3T3-L1 preadipocytes with either pre or anti-miRs and evaluated cell cycle proliferation. These results show a significant increase in preadipocyte proliferation by inhibition of miR-205 (Figure 4C). MiR-205 has been shown to target ErbB3 in cancer cells and it is known that ErbB3 is expressed in 3T3-L1 cells [19,20]. In addition to this upon adding the adipogenesis induction medium to 3T3-L1 cells after miRNA modulation and staining for lipid formation at Day 3, we found significantly increased oil red O staining in the miR-205 inhibited group (Figure 4A, 4B). We hypothesize that miR-205 targets ErbB3 in adipocytes and enhances proliferation; this in turn enhances adipogenesis and lipid production in cultured 3T3L-1 cells after induction to differentiate.

## Conclusion

This is the first study to show diet-regulation of obesity-related miRNAs in a mouse model of polygenic susceptibility for obesity and type 2 diabetes. We show for the first time that miR-205 inhibition in 3T3-L1 preadipocytes increases cell proliferation and lipid accumulation after adipogenic induction.

## Figures and Tables

**Table 1:** HPO diet up-regulated miRs in visceral adipose of NZ10 mice.

microRNA ID	NZ10 HPO/CD	SWR HPO/CD
mmu-miR-100	4.3	0.8
mmu-miR-101a	3.5	1.3
mmu-miR-101b	3.4	0.6
mmu-miR-106a	7.5	1.2
mmu-miR-10a	6.8	1.0
mmu-miR-10b	7.5	1.2
mmu-miR-29c	4.4	0.5
mmu-miR-140*	5.4	0.6
mmu-miR-141	54.1	1.1
mmu-miR-143	2.2	1.2
mmu-miR-148a	20.3	0.9
mmu-miR-148b	4.6	0.6
mmu-miR-182	37.1	0.9
mmu-miR-187	3.7	1.4
mmu-miR-194	4.7	0.7
mmu-miR-200a	89.3	0.8
mmu-miR-200b	173.6	1.0
mmu-miR-200c	1555.7	1.1
mmu-miR-203	2.5	0.5
mmu-miR-204	26.8	1.2
mmu-miR-205	52.3	0.8
mmu-miR-375	145.7	1.0
mmu-miR-429	80.8	1.0
mmu-miR-542-5p	8.5	0.7
mmu-miR-665	4.9	2.1
mmu-miR-691	3.6	2.2
mmu-miR-744	34.6	4.5

**Table 2:** HPO diet down-regulated miRs in visceral adipose of NZ10 mice.

microRNA ID	NZ10 HPO/CD	SWR HPO/CD
mmu-miR-1	−3.4	−1.66
mmu-miR-122a	−3.71	1.0
mmu-miR-199b	−3.93	−1.42
mmu-miR-215	−6.81	9.9
mmu-miR-341	−3.45	−2.5
mmu-miR-342	−3.68	1.4
mmu-miR-379	−3.37	1.4
mmu-miR-483	−5.29	−2.5
mmu-miR-696	−4.16	−3.33
mmu-miR-702	−4.54	−5.0
